# Effects of school-based physical activity on mathematics performance in children: a systematic review

**DOI:** 10.1186/s12966-019-0866-6

**Published:** 2019-11-21

**Authors:** S. Sneck, H. Viholainen, H. Syväoja, A. Kankaapää, H. Hakonen, A.-M. Poikkeus, T. Tammelin

**Affiliations:** 1 LIKES Research Centre for Physical Activity and Health, Rautpohjankatu 8, 40700 Jyväskylä, Finland; 2 Faculty of Education, University of Oulu, Oulu, Finland; 3 Department of Education and Psychology, University of Jyväskylä, Jyväskylä, Finland; 4 Faculty of Education and Psychology, University of Jyväskylä, Jyväskylä, Finland

**Keywords:** Physical activity, Mathematics performance, Intervention

## Abstract

**Background:**

The benefits of physical activity (PA) on children’s health and wellbeing are well established. However, the benefits of PA on academic performance and particularly on mathematics performance warrant systematic analysis. Mathematics is one of the core subjects in school education globally.

**Methods:**

We systematically searched, analysed and synthesized the literature on the effects of school-based PA interventions on mathematics performance in children aged 4–16. A total of 29 studies consisting of randomised trials and other interventions with control groups were identified through a systematic search, and 11 of them provided sufficient data and appropriate design for a meta-analysis.

**Results:**

Of the 29 studies involving 11,264 participants, positive overall effects of a PA intervention on mathematics performance were found in 13 studies (45%) and neutral overall effects in 15 studies (52%). Only one study reported a significant negative result for a subgroup of children in the first half of the intervention. In a risk-of-bias assessment, 12 studies had low, 17 moderate, and none had a high risk of bias. The meta-analysis of 11 studies suggested an overall small positive effect (ES = 0.23) of the interventions. Only one study in the meta-analysis indicated a negative effect in one of the intervention groups.

**Conclusions:**

Adding PA to the school day may enhance children’s mathematics performance or has no negative effects on performance. Several types of PA interventions can be recommended to be added to the school day.

## Introduction

Physical activity (PA) is defined as any bodily movement produced by skeletal muscles that results in energy expenditure [1]. There is extensive evidence indicating that participating in PA is associated with a variety of benefits for children and adolescents, including better physical health [1, 2] better cognitive and mental health [3], a more positive physical self-concept [4], enhanced global self-esteem [4], and improved academic outcomes [5, 6]. Furthermore, higher PA levels in adolescence have been shown to be positively related to the number of years of post-compulsory education and long-term labour market outcomes [7], which translate into both personal and societal benefits.

Worryingly, however, increasing numbers of school-aged children spend a high proportion of their time in sedentary activities, both at school and during their free time [8]. Physical education (PE) lessons tend to constitute the only occasions providing organized PA during the school day, and it is argued that the role of PA during the school day has not been sufficiently promoted in most countries [9, 10]. Somewhat different criteria are used internationally to measure PA, but a common finding is that the amount of PA during the school day is typically small. Globally, less than 20% of children on average are physically active for the recommended 60 or more minutes per day [11, 12]. Less than half of children in the US meet the guidelines of 30 min of PA during a school day [13].

Childhood inactivity has been shown to have detrimental effects not only on children’s physical and mental health but possibly also on their cognitive and academic performance [5, 14]. To respond to the current low levels of PA among children, interventions have been conducted in the past two decades in several European countries, North America and Australia to increase the amount of PA during the school day. The interventions have not only modified children’s cardiovascular disease risk factors [15] but increasing evidence indicates that PA interventions do not have negative effects on children’s academic performance, cognitive function or on-task behaviour and may even benefit academic performance, particularly in mathematics [6, 16, 17].

Several mechanisms or mediating factors may underlie the effects of PA on academic performance among children. Human and non-human brain research suggests that PA has both acute and lasting effects on the structure and function of the central nervous system, and PA is hypothesized to promote children’s development via effects on brain systems that underlie cognition and behaviour [18–20]. There is evidence indicating that PA affects cognition by, for example, influencing the management of energy metabolism and synaptic plasticity [21].

Recent studies support the assumption that PA may affect executive functions [22, 23]. Executive functions involve inhibition, working memory and cognitive flexibility [24], which in turn have been found to be associated with achievement in both reading and mathematics [25]. Several intervention studies have indicated that PA during the school day is positively associated with increased attention and time-on-task [26, 27]. It is also acknowledged that PA can improve children’s cognitive, emotional and behavioural school engagement [28] and thus affect achievement positively. However, the findings on links between PA interventions and cognitive performance in children are still relatively rare and inconsistent [6, 16, 29].

Children’s motor development and related cognitive learning may be another mediating mechanism explaining the positive effects of PA on academic performance. This is suggested by studies showing that children’s physical growth, motor development and cognitive development are closely linked [30–32]. Many cognitive skills, such as visuospatial skills, rapid *automatized* naming and memory skills, contribute to arithmetic learning [33, 34]. Peng and colleagues [35] suggest that deficits in processing speed and working memory are across-age salient cognitive markers of mathematical difficulties. Memory and processing skills might be influenced when PA is added to mathematics instruction or to the school day. For instance, Mullender-Wijnsma and colleagues [36, 37] used repetition and memorization strategies to promote numerical processing speed in their PA intervention study.

It has also been demonstrated that emotional experiences are linked to mathematical achievement [38]. Sorvo and colleagues [39] reported that children as young as eight may experience anxiety about mathematics-related situations and about failure in mathematics. Therefore, including PA in mathematics lessons may affect emotional experiences and thus benefit children’s mathematics performance.

Mathematics is one of the core curriculum subjects, and the role of mathematical skills in modern technological societies is unquestionable [40]. However, in the past decade, concerns about children’s declining interest and performance in mathematics have been expressed internationally [41–43]. Children’s low interest in mathematics may be partially because mathematics is a subject in which students are reported to spend up to 76% of lesson time in sedentary work [10]. If increasing the amount of PA during math lessons or the school day proves to yield higher engagement, interest and enjoyment and in turn contributes to better mathematics performance, a strong argument could be made to introduce more daily PA in schools. To the best of our knowledge, reviews investigating the effects of school-based PA on academic performance in general have been conducted, but this is the first review specifically investigating the effects of PA on mathematics performance.

The aim of this systematic review and meta-analysis is to address the following questions: (a) Do school-based PA interventions have an effect on children’s mathematics performance? (b) What are the characteristics of PA interventions with positive effects on math performance?

## Methods

**Fig. 1:**
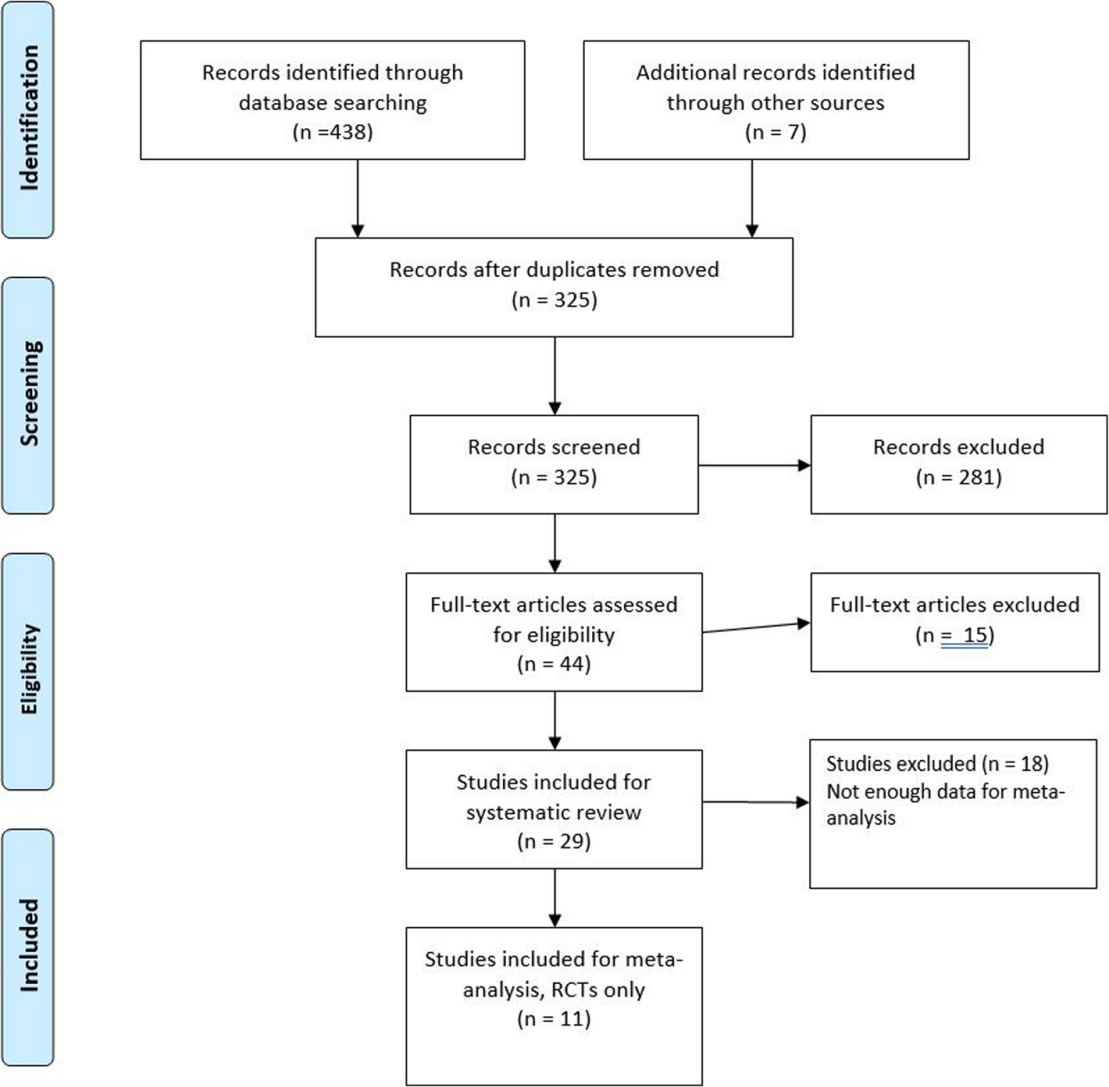
Preferred reporting items for systematic reviews and meta-analyses (PRISMA) study selection flow diagram

We followed the Preferred Reporting Items for Systematic Reviews and Meta-Analysis (PRISMA) guidelines in conducting and reporting on this systematic review. The study selection flow is presented in Fig. 1.

### Eligibility criteria

We used the population, intervention, comparison, outcomes (PICO) model to set the eligibility criteria for the systematic review [44]. **Population:** Intervention participants were 4–16 years old. Studies investigating subgroups of children were accepted (e.g. overweight children). **Intervention:** Controlled trials or other pretest–posttest experiments were included. Both between-group and within-subject designs were accepted. The studies investigated the effects of added school-based (or preschool-based) PA on children’s mathematics performance. PA took place immediately before, during or after school lessons or at break time or was in the form of PE lessons. **Comparison:** Only studies with baseline measurements and control groups were included. **Outcomes**: Studies using scores from standardized or norm-referenced, basic arithmetic or curriculum-based mathematics tests were accepted. **Types of study reports:** Peer-reviewed full-text academic journal articles published in English between January 2000 and November 2018 were examined.

### Study selection and data collection

We searched ProQuest, PsycINFO, SPORTDiscus and Medline in January 2018 for studies to include in this review. The following search terms were employed in Medline: (‘math*’ or ‘arithmetic*’ or ‘numeracy’) AND (‘physical activit*’ or ‘exercise’) AND (‘school*’). The same keywords, slightly modified to adapt to those typical for the search engine, were used in the other databases. An additional search was completed in November 2018 using the same strategy. The complete search strategy details are presented in detail in Additional file 1.

Altogether, we identified 438 studies through the database searches; 325 were retained after removing 113 duplicates. An additional seven studies meeting the inclusion criteria were found through a search of previous systematic reviews on related topics [6, 45] or through searching the reference lists of studies already included. The titles and abstracts of the remaining 325 articles were screened by SS, HV, A-MP and TT. Disagreements were resolved through discussion. Based on consensus decisions, 44 full-text articles were included in the next step. This involved an examination of the full-text articles by SS and HS before finally selecting 29 full-text articles. See the flow diagram in Fig. 1. The main reasons for excluding studies during the process were: 1) No baseline measurement for math performance was conducted 2) The interventions did not have a control group 3) Math performance was measured by teachers’ report cards. Detailed data from the included articles were extracted into Microsoft Excel by SS and HS (Table 1). Where available, pretest–posttest group means were collected to conduct a meta-analysis. Ten original authors were contacted by e-mail to acquire missing data for the analysis. The original authors were given three weeks to reply and were reminded once about the request. Supplementary data were received from three authors. Sufficient data for a full meta-analysis were available for 11 studies.

**Table 1:** Study designs, characteristics and main results

Author and country	Design	Intervention	n	Age (y) or grade	Duration	Type of PA	Achievement Measure(s)	Achievement Outcome(s)	Main Result
*Long-term Interventions*									
Beck et al. (2016), Denmark	Cluster RCT	Motor-enriched mathematics lessons 1) Fine motor math (FMM) 2) Gross motor math (GMM)	165	7.5	6 weeks	Integrated/ specific movement	Standardised Danish test; arithmetic and geometry	GMM improved from T0 to T1. At T2 no significant differences between groups	0
Da Cruz (2017), USA	Group randomised trial	Three 60 min MVPA activities/ week	167	12.1	17 weeks	After school PA	Woodcock-Johnson Test of Achievement III; math fluency and math applied problems	Intervention group had significantly higher math fluency performance but not math applied problems than the control group	+/0
Davis et al. (2011), USA	RCT	After school exercise program 5d/week: 1) Low-dose, 20 mins of PA 2) High-dose, 40 mins of PA	171	9.3	13 weeks	After school PE	Woodcock-Johnson Test of Achievement III, broad mathematics clusters	Significant benefit of exercise on mathematics achievement	+
Donnelly et al. (2009), USA	Cluster RCT	PA across the curriculum 90 mins weekly during academic lessons	203	7–8	3 years	Integrated PA	Standardised Wechsler Individual Achievement Test	Significant improvement in intervention group math scores compared to the control schools	+
Donnelly et al. (2017), USA	Cluster RCT	55 min/week of PA across curriculum (target 100 min), two 10-min lessons per day	584	8.1	3 years	Integrated PA	Standardised Wechsler Individual Achievement Test	No significant impact of intervention on math achievement	0
Elofsson et al. (2018), Sweden	Pre-test vs post-test mixed factorial design	Two 30 mins PA sessions/week: Math in action (MIA) group and Common numerical activities (CNA). Control group.	53	5.8	3 weeks	Integrated PA	Verbal arithmetic test	MIA -group developed significantly more than students in control group with respect to counting forward, naming numbers, number line estimation, verbal addition and subtraction	+
Erwin et al. (2012), USA	Quasi-experimental study	Daily 20 min PA breaks with math content in class.	29	8.9	20 weeks	PA breaks with math content	Curriculum based math fluency 1 min test and stadardised tests	Intervention students had significantly higher curriculum based math scores than the controls but not standardised tests or grades	+
Fedewa et al. (2015), USA	Cluster RCT	PA breaks throughout the day, 5 min at once, 20 min/day	460	grades 3–5	8 months	PA breaks with math content	National standardised Measures of Academic Progress (MAP)	Treatment classrooms experienced larger gains in mathematics achievement	+
Hraste et al. (2018), Croatia	RCT	4 x PA integrated into 45 min mathematics/geometry lessons	36	10.4	1 week	Integrated PA	A standard test for assessing mathematical knowledge and a geometry test	Experimental group was significantly more successful in the geometry tests than the control group	+
Gao et al. (2013), USA	Crossover design	30 min of aerobic activities or exergaming (dance) three times a week	208	10–12	2 years	PE lessons	Math scores for the Utah Criterion-Referenced Test	Children in PA intervention showed more improvement on math scores in year 1 and 2 than comparison groups	+
Katz et al. (2010), USA	Intervention	Multiple type structured PA breaks throughout the school day (> 30 mins/day)	1214	7–9	8 months	PA break in class	Missouri Academic Peformance (MAP) scores	No significant differences between intervention and control groups in MAP scores of mathematics performance	0
Lubans et al. (2018) Australia	Cluster RCT	Maximising students’ MVPA during PE classes through PE teacher in-service training	1173	12.9	7 months	Higher intensity PE lessons	National Assessment Program -Literacy and Numeracy (NAPLAN): understanding, fluency, problem solving, and reasoning in numeracy	A small to medium effect on mathematics performance. The effect was equal to approximately one quarter of the increase in typical age-level mathematics performance.	+
Mavilidi et al. (2018), Australia	Cluster RCT	15 min learning session once per week. Four intervention groups: 1) PA related to the learning tasks, 2) observation of peers’ PA 3) PA unrelated to the learning task 4) control condition	120	4.7	4 weeks	Integrated PA + PA breaks	Counting, number line estimation, block counting, numerical magnitude, comparison, numerical identification	The performing integrated PA condition performed significantly better than the other conditions, with the largest effect on number line estimation and numerical magnitude comparison	+
Mead et al. (2016), USA	Pretest–posttest design	1) Two 5-min PA breaks during each math period (ACTB), 2) Students always sat on stability balls (STAB)	81	11–12	1 year	Stability ball and PA break	Standards-based Minnesota Comprehensive Assessments (MCA) and Measures of Academic Progress (MAP)	PA breaks were not effective in improving math scores but use of stability balls was effective	0
Mullender-Wijnsma et al. (2015), Netherlands	Quasi-experimental design	Physically active academic lessons 3x week. Each lesson had 10–15 min math problems and 10–15 min language problems	228	8.1	21 weeks	Integrated PA	Tempo Test Rekenen (Speed arithmetic test)	3rd grade intervention group scored significantly higher on mathematics compared to the controls while the 2nd grade group scored significantly lower than the controls	+/−
Mullender-Wijnsma et al. (2016), Netherlands	Cluster RCT	Physically active academic lessons 3x week. Each lesson had 10–15 min math problems and 10–15 min language problems	499	8.1	2 yrs. (22wk/year)	Integrated PA	Two standardised math tests (speed and general math skills)	Intervention group had significantly greater gains in mathematics speed test and general mathematics	+
Resaland et al. (2016), Norway	Cluster RCT	90 min/week physically active lessons (30 min math), 5 min/day PA breaks, 10 min/day PA homework	1129	10	7 months	Integrated PA + PA breaks+ PA homework	Standardised Norwegian national tests	No significant intervention effect	0
Resaland et al. (2018), Norway	Cluster RCT	90 min/week physically active lessons (30 min math), 5 min/day PA breaks, 10 min/day PA homework	1129	10	7 months	Integrated PA + PA breaks+ PA homework	Standardised Norwegian national tests	Boys and girls in the low performing tertile had a beneficial trend. Middle and high performing girls responded with negative trends.	0
Riley et al. (2016), Australia	RCT	Movement based learning in mathematics 3 × 60 min/week.	240	11	6 weeks	Integrated PA	Standardised mathematics progressive achievement test	No significant effect on mathematical performance	0
Sallis et al. (1999), USA	Experimental design	Three days/week, 30 min lessons of 1) Specialist-taught program 2) Trained classroom teacher taught program	754	9	2 years	Extra PE lessons	Norm-referenced Metropolitan achievement test	More time in PE did not have harmful effects on math achievement test scores	0
Sjöwall et al. (2017), Sweden	Intervention	180 min extra PA/ week	470	6–13	2 years	Extra PA activities	Arithmetic test: One min addition and subtraction	No significant effects were found for arithmetic	0
Snyder et al. (2017), USA	Intervention	Students active for at least 50% of the 70-min mathematics lesson	24	3rd grade	5 weeks	Integrated PA	Common Summative Assessment (CSA)	No statistically significant differences between the two classrooms	0
Tarp et al. (2016), Denmark	Cluster RCT	60 min daily PA a) PA integrated into academic subjects b) Recess PA activities C) PA homework daily D) active transportation	632	12–13	20 weeks	Integrated + recess PA + PA homework + active transportation	Custom made grade specific mathematics tests: arithmetic, algebra, problem-solving and geometry	No significant effect of the intervention on mathematics skills	0
Watson et al. (2018,) Australia	Cluster RCT	3 × 5 min active breaks in classroom daily, five times a week	312	9.1	6 weeks	PA break in class	Westwood one minute test on basic number facts (subtraction subtest)	No intervention effect on mathematics	0
Vetter et al. (2018), Australia	Randomized crossover trial	Three 20 min sessions/week, physically active learning of numeracy skill of times Tables (TT).	85	9.8	6 weeks	Integrated PA	Custom made curriculum based TT test and general standardised assessment from the Australian National Assessment Program-Literacy and Numeracy (NAPLAN)	No significant difference in the TT test between PA and control conditions. Significantly greater improvement in general numeracy for PA group than control group	0/+
*Acute Effects*									
Harveson et al. (2018), USA	Randomized crossover design	a) AE, 30 min aerobic exercise, b) RE, 30 min resistance exercise, c) NE, no exercise.	91	15.9	Three separate sessions separated by 7 days each	PA session before test	Battery of four 10-question math tests taken from New York State Testing Program exams	Acute RE and AE did not significantly improve scores on a mathematics test	0
Howie et al. (2015), USA	Within-subjects randomised experiment	PA breaks: 1) 5 min 2) 10 min 3) 20 min breaks or 10 min sedentary lesson	96	10.7	One consistent time of day and week	PA breaks in class	One min math fluency test based on state curriculum standards	Math scores were higher after the 10-min and 20-min exercise breaks compared with the control, but not after the 5-min exercise break	+
Phillips et al. (2015), USA	Within -subjects design, repeated measures design	20 min vigorous PA followed by a math test at 30 min and 45 min	72	14–15	One session	PA session before test	The New York State Testing Program: a) number sense and operations, b) algebra, c) geometry and d) measurement; 4 five-minute tests	Students achieved 11–22% higher math scores at 30 min post PA compared with other time points	+
Thompson et al. (2016, USA)	Cluster RCT, between-groups design	Special 40 min PE class (min 20 of MVPA) directly before math test	791	10–11	One session	PA session before test	standardised Northwest Evaluation Association Measures of Academic Progress	No statistically significant differences in change in math results	0
Travlos (2010), Greece	Experiment, within-subjects desing	Interval aerobic run (four sets of 4-min run) PE lesson-before math test.	48	13–15	Four days	PA session before test	Simple addition problems; two min-speed and accuracy test	Numerical speed and accuracy of students who attended the first, third, and fifth hour of the daily lessons increased, but there was a decrease in the sixth-hour lesson	+

+ = positive effect; 0 = no effect; − = negative effect. *MVPA* moderate to vigorous physical activity, *PA* physical activity, *RCT* randomized controlled trial, *PE* physical education

### Risk-of-Bias assessment

A risk-of-bias assessment of the final sample of 29 studies was conducted using combined, modified criteria previously used by Lonsdale [46] and Van Sluijs [47] and following the guidelines of the Cochrane Handbook for Systematic Reviews of Interventions [44]. Some slight modifications were made to the risk assessment criteria to adapt to experiments conducted in the fields of education and psychology. It is acknowledged that experimental and quasi-experimental designs to evaluate the effects of policy and programs need to have adequate statistical power to detect meaningful size impacts. Therefore, the criterion of power calculation was added to the assessment [48]. Each study received ‘0’ (does not meet the criterion) or ‘1’ (meets the criterion) for each criterion based on an analysis of the reporting in the original article.

### Meta-analysis procedures

Only randomized controlled trials were included in the meta-analysis to ensure high-quality interpretation [44]. Effect size (ES) estimates were calculated using Cohen’s d. Only post-intervention (not mid-intervention) mean (M) values were used in the analysis. For between-group designs, Cohen’s d was calculated as follows:

$$ d=\frac{\left({M}_{treatment}^{t2}-{M}_{treatment}^{t1}\right)-\left({M}_{control}^{t2}-{M}_{control}^{t1}\right)}{SD_{pooled}}, $$

where ${M}_{treatment}^{t1},{M}_{treatment}^{t2},{M}_{control}^{t1}\ \mathrm{and}\ {M}_{control}^{t2}$ are the baseline (t1) and post-intervention (t2) means in the treatment and control groups, and *SD*_*pooled*_ is the pooled standard deviation.

**Fig. 2:**
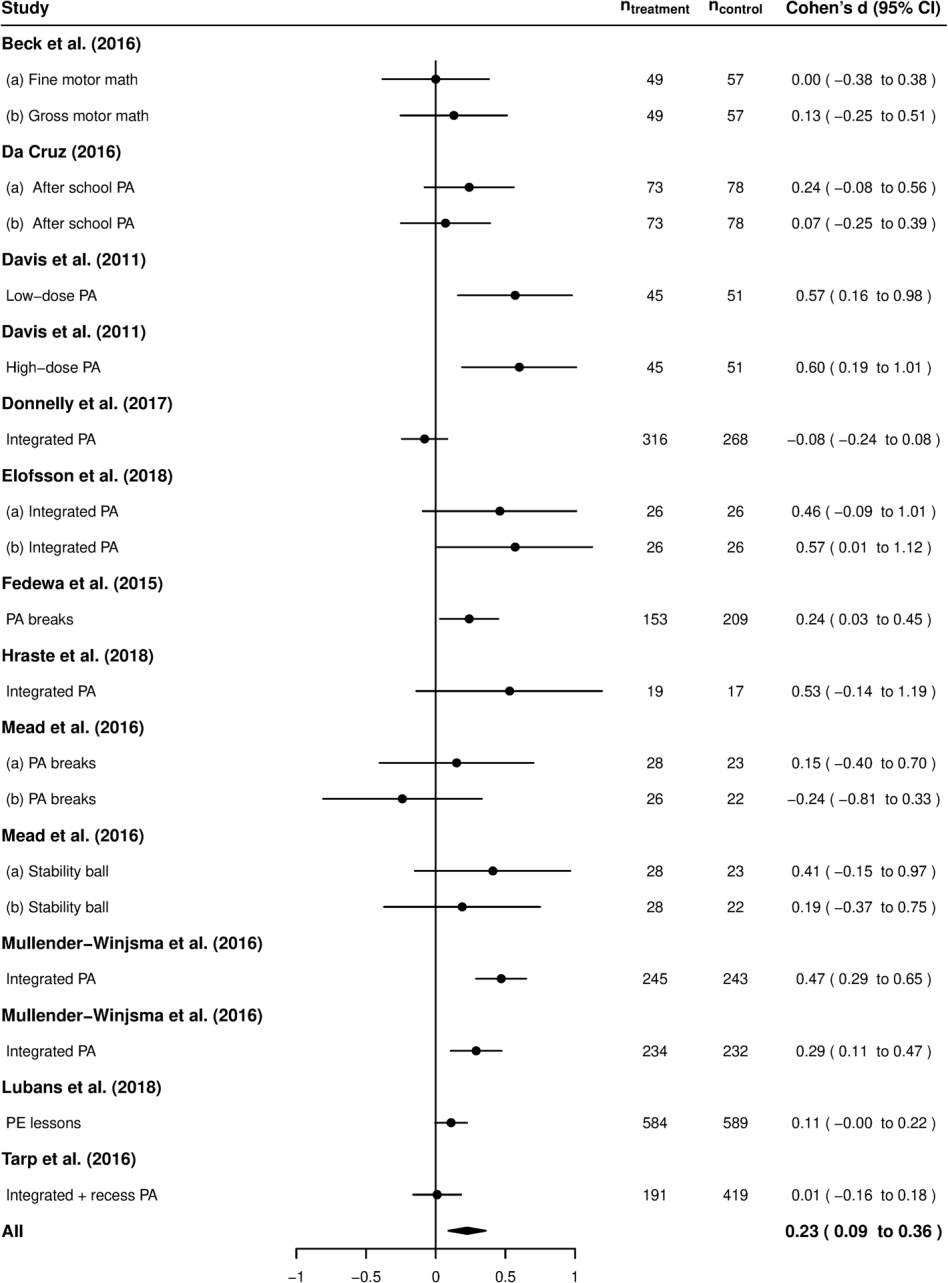
Forest plot. Pooled ES estimates and 95% confidence intervals were calculated using a random effect model. ● Individual study effect sizes were calculated using Cohen’s d. ♦ Summary effect size

The I2 statistic was calculated [49] to evaluate heterogeneity among the studies, and the following values were used for interpretation: < 30%, mild; 30–50%, moderate and > 50%, high heterogeneity [50]. Pooled ES estimates and 95% confidence intervals were calculated using a random effect model. The ES estimates and confidence intervals of individual studies and pooled estimates are presented in Fig. 2. A decision was made to consider ES ≥ 0.8 large; ≥ 0.5 medium and ≥ 0.2 small [51, 52]. As heterogeneity was found to be large in the sample of 11 studies, a moderator analysis was performed. Meta-regression analyses were conducted to assess the relationship between ES estimates and the following study-level variables: participants’ age and duration and type of intervention. Further analyses were conducted using the statistical software package R (version 3.4.3). The 95% confidence intervals for the ES of individual studies, heterogeneity and meta-regression estimates were calculated using the MBESS package [53].

## Results

### Systematic review of study characteristics

A total of 29 intervention studies were included in the systematic review. A descriptive summary of the characteristics of the reviewed studies is presented in Table 1. The countries of origin of the studies are as follows: USA [14], Australia [5], Denmark [2], the Netherlands [2], Norway [2], Sweden [2], Croatia [1] and Greece [1]. The participants ranged in age from 4.7 to 16 years old. Two of the studies were conducted in a preschool setting [54, 55]. The total number of participants in the intervention and control groups ranged from 29 to 1214 children [56, 57]. The intervention participants comprised 11,264 children.

Standardized or national-level mathematics tests were employed in 22 studies to measure mathematics learning outcomes. The remaining studies employed custom-made tests that typically assessed basic arithmetic skills or were based on local age-level curriculum goals. Many studies employed more than one type of mathematics test [36, 56, 58–61]. The length of the interventions varied between 1 week [62] and 3 years [63, 64]. Of the 29 studies, 5 investigated the acute effects of PA interventions, that is, a short PA session lasting 5–40 min took place right before a mathematics test.

The content of the interventions varied greatly. In 11 studies, PA was integrated into mathematics lessons and included curriculum-based mathematics goals [10, 36, 37, 40, 54, 55, 62–66]. Positive results were reported in 5 (45%) of these 11 studies [36, 54, 55, 62, 63]. Only one study reported significant negative results [37] for a subgroup of 8-year old children in the first half of the intervention. Two studies reported partly positive and partly neutral results [40, 66].

In five studies, the intervention consisted of extra PE lessons, more intense PE lessons or other extra teacher-led PA during the school day [58, 67–70]. Three out of five interventions showed positive results on mathematics performance [68–70], while one study reported partly positive and partly neutral results [58]. One of the studies reported neutral effects [67]. It is noteworthy that some of these studies involved subgroups; da Cruz [58] studied only girls, Gao and colleagues [70] studied only Latino children and Davis and colleagues [68] studied only overweight children.

Five intervention studies involved short PA breaks during lessons or in the middle of the school day [56, 57, 59, 71, 72]. The length of PA breaks varied between 5 min and 20 min, and there could be several breaks during a day. Two of the five studies indicated positive results [56, 71] and the rest reported neutral effects.

The remaining three long-term interventions used a combination of various types of PA [60, 73, 74]. The interventions included, for example, PA breaks, integrated PA, active transportation to school and PA homework. These interventions had no overall effect.

In four of the five studies investigating the acute effects of PA on math performance, PA sessions took place right before math performance testing sessions [61, 75–77]. The PA sessions lasted approximately 20–30 min and varied in intensity. Two of these studies indicated positive effects of PA sessions on math scores [61, 76] and two indicated neutral effects [75, 77] One of the acute effect studies employed 5–20-min breaks during math lessons [9]. In this study, math scores proved to be higher after 10- and 20-min exercise breaks but not after 5-min breaks. See Table 1 for all the details.

In some of the reviewed PA interventions, additional findings were reported for subgroups of participants. Howie, Schatz and Pate [9] reported that classroom exercise breaks had a positive effect on mathematics scores for participants with lower IQ, higher aerobic fitness or lower school engagement. Beck and colleagues [40] reported that average mathematics performers (not low performers) benefited from math-related gross motor activities but not from fine motor activities. In a large Norwegian study [73] (*n* = 1129), subgroup analysis indicated positive intervention effects for pupils with the poorest math baseline scores. In a later analysis, a negative trend (not a significant effect) in mathematics performance was found for middle and high-performing girls [74]. In a study by Sjöwall [67] subgroup analyses revealed no favourable intervention effects for children with low baseline fitness or cognition.

### Results of meta-analysis

Data for meta-analysis were available for 11 studies. Some of these studies included two different intervention conditions and/or two separate mathematics outcomes, thus leading to two to four different ES estimates for these studies. The results of the analysis are presented in Fig. 2. Small ESs (0.2 ≤ ES ≤ 0.5) were detected in six intervention studies. Moderate ESs (≥ 0.5) were found for four interventions. One of the interventions indicated a small negative ES (− 0.24) [59]. The rest indicated no effect (− 0.2 ≤ ES < 0.2). Overall, on average a small positive effect (d=0.23) was found for all the interventions. The level of statistical heterogeneity between the intervention groups was high, I^2^ = 69.6%. In the moderator analysis, the age of participants (β = − 0.051, *p* = 0.045) and the duration of intervention (β = − 0.003, *p* = 0.002) were found to explain heterogeneity. The type of intervention was not found to influence ESs. See Table 2 for a full analysis.

**Table 2:** Moderator analysis

	β	SE	t-value	df	*p*-value	95% CI Lower	95% CI Upper
Age	−0.051	0.023	−2.21	13	0.045	− 0.100	− 0.001
Duration of intervention	− 0.003	0.001	−3.71	13	0.002	−0.005	−0.001
Type of intervention:							
Extra PA	0.090	0.138	0.652	13	0.525	−0.208	0.388
Integrated PA	0.015	0.146	0.098	13	0.923	−0.302	0.331
PA breaks	−0.115	0.120	−0.960	13	0.355	−0.373	0.144
Other	−0.108	0.113	−0.950	13	0.359	−0.353	0.137

### Results of risk-of-Bias assessment

The results of the risk-of-bias assessment analysis are shown in Table 3. Of the 29 studies, 12 were rated as having a low risk of bias (> 67% of total score) and 17 were rated as having a moderate risk of bias (between 33 and 67% of the total score). None of the studies was rated as having a high risk of bias. Only eight studies reported power calculations to determine sufficient sample sizes. Of those reporting positive effects of PA on mathematics performance, power calculations were provided in five [36, 58, 61, 64, 74].

**Table 3:** Criteria were rated “1” if evidence was found in the article. Within-subjet designs: No power calculation required.

	1. Randomisation	2. Baseline Comparable	3. Baseline Values Accounted for in Analyses	4. Timing	5. Blinding of Measuring	6. Validated Outcome Measures	7. Dropout Analysis	8. Reporting of Results	9. Power Calculation	Total Score
*Between-groups designs*										
Beck et al. (2016)	1	1	1	1	0	1	1	0	0	6/9
Da Cruz (2017)	1	1	1	1	1	1	1	1	1	9/9
Davis et al. (2011)	1	1	1	1	1	1	1	0	0	7/9
Donnelly et al. (2009)	1	1	1	1	1	1	1	1	1	9/9
Donnelly et al. (2017)	1	1	1	1	1	1	1	1	1	8/9
Elofsson et al. (2018)	1	1	1	1	0	0	0	1	0	5/9
Erwin et al. (2012)	0	1	1	1	0	1	0	1	0	5/9
Fedewa et al. (2015)	1	0	1	1	0	1	0	1	0	5/9
Gao et al. (2013)	0	0	1	1	0	1	1	1	0	5/9
Hraste et al. (2018)	1	1	1	1	0	1	0	1	0	6/9
Katz et al. (2010)	1	1	1	1	0	1	0	1	0	6/9
Lubans et al. (2018)	1	1	1	1	1	1	1	1	1	9/9
Mavilidi et al. (2018)	1	1	1	1	0	0	0	1	0	5/9
Mead et al. (2016)	1	1	1	1	0	1	1	1	0	7/9
Mullender-Wijnsma et al. (2015)	1	0	1	1	0	1	1	1	0	6/9
Mullender-Wijnsma et al. (2016)	1	0	1	1	0	1	1	1	1	6/9
Resaland et al. (2016)	1	1	1	1	0	1	1	1	1	7/9
Resaland et al. (2018)	1	1	1	1	0	1	1	1	0	7/9
Riley et al. (2016)	1	1	1	1	0	1	1	1	1	7/9
Sallis et al. (1999)	1	0	1	0	0	1	0	1	0	4/9
Sjöwall et al. (2017)	0	1	1	1	0	0	1	1	0	5/9
Snyder et al. (2017)	0	1	1	1	0	0	0	1	0	4/9
Tarp et al. (2016)	1	1	1	1	0	0	1	1	1	6/9
Thompson et al. (2016)	1	1	1	1	0	1	0	1	0	6/9
Watson et al. (2018)	1	1	1	1	0	1	0	1	0	6/9
Vetter at al. (2018)	1	1	1	1	0	1	0	1	0	6/9
*Within-subjects design*										
Harveson et al. (2018)	1	1	1	1	0	1	0	1		6/8
Phillips et al. (2015)	1	1	1	1	0	1	1	1		7/8
Howie et al. (2015)	1	1	1	1	0	0	1	1		6/8
Travlos (2010)	0	0	1	1	0	0	1	1		4/8
SUM OF ALL STUDIES	25	24	30	29	5	23	18	28		

1. SELECTION BIAS. Randomization of conditions (cluster randomization accepted). 2. SELECTION BIAS. Baseline comparable on key characteristics. 3. PERFORMANCE BIAS. Baseline values accounted for in the analyses. 4. PERFORMANCE BIAS. Timing of intervention and measurements. 5. PERFORMANCE BIAS. Blinding of measuring. 6. DETECTION BIAS. Validated measures of mathematics outcome(s) used. 7. ATTRITION BIAS. Dropout not more than 20% for interventions < 6 months and not more than 30% for > 6 months. 8. REPORTING BIAS. 9. OTHER BIAS. Power calculation performed

## Discussion

The purpose of this systematic review was to examine the effects of school-based PA interventions on children’s mathematics performance and to detect and identify the features of effective interventions. The review indicated that 45% of the 29 intervention studies included in the analysis based on a rigorous literature search showed positive effects, and the meta-analysis of 11 studies suggested an overall small positive effect of school-based PA interventions on children’s mathematics performance. Only one study indicated significant negative effects in a subgroup of participants. Taken together, the results of this review provide evidence to support the assumption that increasing school-based PA can have positive effects on children’ mathematics performance and that it does not have harmful effects on performance. The findings seem to be in line with earlier reviews investigating academic performance in general [6, 16, 17].

The moderator analysis revealed that older age of participants and longer duration of intervention were negatively associated with ESs. This suggests that younger children may benefit more from PA interventions than older children and that longer interventions are not necessarily more effective than shorter ones.

This review included various types of PA interventions —physically active mathematics lessons integrating PA into academic learning goals, the introduction of PA during or after school, adding short PA breaks during academic lessons or in the middle of the school day and bursts of activity right before mathematics testing. There was no clear evidence indicating that some of the types of PA would be more effective than the others. However, increasing the amount of traditional PE lessons did not seem to have a positive effect on mathematics learning, whereas PE lessons with more intense PA did make a difference. In their earlier review and meta-analysis Alvarez-Bueno and colleagues [17] concluded that curricular PE lessons seemed to be the most appropriate type of PA to improve children’s academic achievement, although integrating PA in classroom lessons also benefited mathematics-related skills. Hence, drawing conclusions on what type of PA works best remains a challenge.

Subgroup analyses showed that students’ cognitive abilities may have an effect on how much they benefit from increased PA with respect to math performance gains. Two studies [9, 74] suggested trends implying that children with lower IQ or baseline achievement and low school engagement may benefit more from PA interventions than other participants. Nonetheless, Beck and colleagues [40] reported conflicting results. Studies focusing on overweight children [68] and children with a minority background [70] reported some positive effects of PA on math performance. Although interpretations need to be made with caution, the analyses suggest that children experiencing barriers to learning might benefit more from increased amounts of PA in school than other children.

The findings of acute effect studies by Phillips and colleagues [61] and Travlos [76] indicated that the timing of PA during the school day may be important, thus providing support for the view that the placement of PA breaks before cognitively challenging tasks may be beneficial. The results of Howie and colleagues [9] suggested that 5-min PA breaks may be too short to have effects on math performance, whereas breaks lasting 10 or 20 min may have beneficial effects.

Many of the reviewed studies included measures of other outcomes, for example, measures of cognitive skills, executive functions, behaviour, brain activation and language achievement. Further examination of these factors in future studies would be helpful in determining how or why PA might affect mathematics performance. For instance, Beck and colleagues [40] have argued that favourable effects of motor activities on academic performance may be accounted for by changes in the visuo-spatial short-term memory and improved attentional resources. In the study by da Cruz [58] participation in a PA intervention was found to be positively associated with changes in both inhibition and mathematics fluency. Davis and colleagues [68] evidenced increased prefrontal cortex activity in study participants and suggested that cognitive changes might be the result of neural simulation rather than being mediated by cardiovascular benefits. The results of a study by Elofsson and colleagues [55] showed that children’s motor skills explained almost 16% of the variation in mathematical measures.

Despite the positive effects shown in almost half of the reviewed interventions, it is unclear whether PA per se was the cause of those positive effects. For instance, Mullender-Wijnsma and colleagues [36] suggested that academic engagement or an innovative teaching method consisting of repetition and memorization techniques might partially explain the positive effects of their PA intervention. Although only designs with an intervention vs. control condition comparison were included in the review, alternative explanations for positive intervention effects cannot be ruled out. That is, it is possible that instead of or in addition to direct effects of increased PA, it was the attention from adults, a change in routine or pedagogical practices and increased engagement and enjoyment that produced the positive results. It is probable that children may experience psychological changes due to the social interaction that occurs during PA sessions [18]. These perspectives require further investigation.

Because some learners associate anxiety or dislike with mathematics lessons, it is relevant to note that some of the interventions integrating PA and mathematics reported positive experiences by teachers and students, and student engagement during lessons was also enhanced [78]. Learning activities that are engaging promote small group social interaction and de-emphasize competition, thus enhancing learning [45].

Three recent large-scale Scandinavian multicomponent interventions [60, 67, 73] where various types of PA were added to the school day did not indicate significant positive effects on mathematics performance. This may be because Scandinavian school days already include regular breaks, weekly PE lessons and a pedagogy that activates children. This raises the question of whether there may be an upper limit after which the increased amount of PA no longer improves academic achievement. Nonetheless, the finding that academic performance is not harmed by additional PA is important because of the beneficial effects of PA on children’s physical and mental health.

One of the strengths of this review is that only studies with baseline measures and control groups were accepted for analysis. Furthermore, only studies using random control trial designs were included in the meta-analysis. Studies with teacher-reported grades without any test scores were not included in the sample, as grades are often tied to local and national educational culture and curriculum. The risk-of-bias assessment was added to the analysis to provide information at the evidence level, and it revealed no studies with a high risk of bias.

There are some limitations to this study that must be noted. The number of high-quality studies on the topic is still low, which posed challenges, particularly as regards the meta-analysis. Missing data in the original articles or deficiencies in study designs reduced the pool of studies eligible for ES analysis, which might affect the strength of the conclusions. The large statistical heterogeneity of the results may reduce the reliability of the meta-analysis and hence the overall effects size should be interpreted with caution. On the whole, heterogeneity of the results may be due to different educational contexts, measures of mathematics performance and nature of PA selected for the intervention. Some methodological challenges were identified in the original studies, such as the lack of power calculations or evaluations of treatment fidelity. The commitment of participants was a factor that compromised interpretations in some of the studies [60]. Even though nationally used curriculum-based tests are needed, future studies should preferably utilize internationally acknowledged tests and task types for basic mathematical skills [56].

Despite the promising results, more replication studies with similar measurement, adequate sample sizes and carefully planned control groups are needed to establish a potential causal relationship between PA and academic performance [16, 29]. Regarding the theoretical basis upon which assumptions about the mechanisms behind the effects of PA can be drawn and tested, a clear need exists for merging neuroscientific, psychological or educational theorizing and concepts to better understand the mechanisms behind the effects of PA on children’s academic performance. More research is needed to answer questions such as the extent to which we can reduce the time spent in sedentary activities and not compromise the academic learning of children.

Schools have a key role in introducing and integrating PA into children’s everyday lives. Accordingly, every opportunity should be explored and made use of in the school curricula and pedagogical practices to diminish the harmful effects of a sedentary lifestyle. The information presented in this review and meta-analysis provides some evidence to back the supposition that adding more PA to the school day and to lessons in the form of PA breaks, extra PA sessions, more intense PE lessons or PA integrated into academic lessons may enhance children’s academic performance and mathematics learning. The results of this systematic review and further studies can help convince educators and policy makers to recommend the addition and effective integration of PA into the school day.

## Supplementary information

**Additional file 1:** Systematic search strategy.

## Data Availability

Most of the data generated or analysed during this study are included in this published article and its supplementary information files, or in the published original articles included in the review. A small amount of data (math test means missing in the included original articles) were received directly from original authors and are available on request from the corresponding author.

## Abbreviations

ES: Effect size
M: Mean value
MBESS: Methods for the Behavioral, Educational, and Social Sciences
MVPA: Moderate to vigorous physical activity
PA: Physical activity
PE: Physical education
